# Process Evaluations for the Scale-Up of Complex Interventions – a Scoping Review

**DOI:** 10.5334/ijic.7600

**Published:** 2024-11-08

**Authors:** Lekha Rathod, Martin Heine, Daniel Boateng, Monika Martens, Josefien van Olmen, Grace Marie Ku, Kerstin Klipstein-Grobusch

**Affiliations:** 1 Julius Global Health, Julius Center for Health Sciences and Primary Care, University Medical Center Utrecht, Utrecht University, Utrecht, The Netherlands; 2 Institute of Sport and Exercise Medicine, Faculty of Medicine and Health Sciences, Stellenbosch University, Cape Town, South Africa; 3 Department of Epidemiology and Biostatistics, Kwame Nkrumah University of Science and Technology, Kumasi, Ghana; 4 Department of Public Health, Institute of Tropical Medicine, Antwerp, Belgium; 5 Department of Family Medicine and Population Health, Faculty of Medicine and Health Sciences, University of Antwerp, Antwerp, Belgium; 6 Division of Epidemiology and Biostatistics, School of Public Health, Faculty of Health Sciences, University of the Witwatersrand, Johannesburg, South Africa

**Keywords:** complex health interventions, scale-up, process evaluation, implementation research, integrated care

## Abstract

**Introduction:**

Complex health interventions (CHIs) are common in (public) health and social care practice and policy. A process evaluation (PE) is an essential part of designing and testing CHIs and questions what is implemented, the mechanisms of change, and how context affects implementation. The scale-up of CHIs is challenging and heterogeneous, making the accompanying PE unique to the nature of the inquiry.

**Methods:**

We conducted a scoping review to describe the current practice of conducting PEs alongside or following the scale-up of CHI. Eight primary data sources were searched and data extracted on study characteristics, intervention characteristics, methods used in relation to the PE, and stakeholders included.

**Results:**

We reviewed 10,538 records and included 56 studies. Seven common thematic areas emerged in which CHIs were being scaled-up. The use of scale-up specific frameworks was rare, and common outcomes of the process evaluation focussed on barriers and facilitators in relation to the context; often obtained “once-off” using qualitative and quantitative data sources. Scale-up strategies reported were: supporting increased coverage, comprehensiveness, and institutionalisation; often simultaneously.

**Conclusion:**

Variations in the conduct of process evaluations during the scale-up phase of complex health interventions may reflect differences in context, conceptual challenges, the multi-dimensional nature of scale-up, and the point of engagement with the health care system (e.g., community-level). Ideally, a process evaluation is a recurrent continuous process, leveraging a systems-driven understanding and triangulation of qualitative and quantitative data, that takes place alongside the scale-up project to inform real-world adaptations of scale-up strategies and (untoward) mechanisms of impact when applicable.

## Introduction

Interventions in health and social care services, public health practice, and other areas of social and economic policy are often complex interventions with consequences for health [1]. Examples of complex health interventions (CHIs) are strengthening integrated care of diabetes and hypertension, home management of malaria, or stroke management at the primary care level through digital health. These interventions can be considered complex because of the properties of the intervention itself, such as: the number of components involved; the range of behaviours targeted; the expertise and skills required by those delivering and receiving the intervention; the number of groups, settings, or levels targeted; or the permitted level of flexibility of the intervention or its components [2,3]. The effects of CHIs can be evaluated from individual level (health) outcomes through to societal level impact and policy [1].

However, for many urgent health needs, the key question is not about testing or developing new CHIs, but rather scaling-up already existing interventions through implementation of evidence-based practices and research findings into clinical practice, i.e., implementation research. When implemented, CHIs tend to be adapted for different contexts to correspond to local practice; this helps evaluators distinguish between adaptations required to fit different contexts and adaptations that may compromise intervention fidelity [2].

A scale-up strategy refers to “the processes and actions to increase the impact of health interventions so as to benefit more people and to foster policy and programme development on a sustainable basis” [4,5]. Scale-up has been conceptualized in three dimensions [4,5,6]: 1) increasing population coverage; 2) expanding the intervention programme; and 3) integration into health system and services.

A process evaluation (PE) is an essential part of designing and testing CHIs and is vital in building an evidence base that informs policy and practice [1,2]. As the PE framework developed by the UK Medical Research Council describes, through conducting a PE, one could obtain a pivotal understanding related to context (contextual factors and causal mechanisms), implementation (fidelity, dose, adaptations, reach), and mechanisms of impact (participant responses, mediators, and unanticipated pathways and consequences) [1,2]. It can also provide policymakers and practitioners with vital information about how the CHI might be replicated in different contexts and knowledge on how to implement it [2]. These different contexts may reflect differences in the external environment (policies/resources) across regions and countries and further help identify factors that shape the implementation (or scale-up) of a CHI by examining the policies, resources, and cultural attitudes that impact the intervention. Furthermore, these contexts may also reflect differences in the internal environment with respect to organizational and patient characteristics, perspectives, and infrastructure, which again, may differ not only across borders but also within borders (e.g., provinces, regions, cities, towns, neighbourhoods) [7,8,9,10,11]. As such, a PE helps to assess whether fidelity to the original CHI design was maintained and ensure that it was implemented as intended, while simultaneously recognizing and observing any pragmatic adaptations that were needed to fit the local context [1,2,7,8]. Thus, the process of scaling-up CHIs is complex and heterogeneous, making the accompanying PE essential yet distinct due to the unique nature of the inquiry (e.g., developing/testing an intervention vs. bringing an intervention to scale). A previous review of process evaluations for clinical trials in hospital settings has underpinned the importance of standardized reporting of PEs and better descriptions of the use of frameworks in those evaluations [12].

Hence, in this scoping review, we aim to describe the current practice of PE in the scale-up of CHIs. Specifically, we aim to examine the key functions of a PE as explained above, but also in terms of the *scaling-up* of a CHI, including methods for conducting the PE, theoretical underpinnings, and stakeholders’ involvement. This review will support a better understanding of the practice and role of PEs in the scale-up of complex interventions, identify trends and challenges in such practices, and inform future developments in the underlying theory and methodology (e.g., conceptual framework for process evaluation alongside scale-up, reporting standards) supporting these evaluations.

## Methods

This scoping review was conducted in accordance with the guidance outlined by Arksey and O’Malley and The Joanna Briggs Institute (JBI), and reported in line with the Preferred Reporting Items for Systematic Reviews and Meta-Analyses (PRISMA) scoping review extension [13,14]. A protocol for this review has been made publicly available through the open science framework [15]. In collaboration with a medical librarian, eight primary data sources (PubMed, Embase, CENTRAL, Web of Science, CINAHL, Global Health, Scielo and African Index Medicus) were searched for PEs in the context of scale-up of CHIs (see Panel 1).

**Panel 1 PubMed search strategy for this scoping review**

The search strategy for PubMed was as follows: {“Process eval*” [tiab] OR “Program eval*”[tiab] OR “Process Assessment, Health Care”[mesh] OR fidelity[tiab] OR dose[tiab] OR reach[tiab] OR variat*[tiab] OR context*[tiab]} AND {“Scale-up”[Title/Abstract] OR “Scaling-up” [Title/Abstract]}.

At the title level, studies not in English *were included* for abstract screening. Subsequently, at the abstract level, articles in non-English languages (e.g., French, Spanish, and Portuguese) were screened using Google Translate, with a native speaker of the languages contacted in case the articles were included (ultimately not required). The search was complemented by backward (screening reference lists) and forward citation hashing (using Google scholar) of included articles to identify articles possibly missed during the initial search. An initial screening of titles was conducted by one of two reviewers (LR or MH) to exclude any articles clearly ineligible (e.g., articles on scale-up in botany, zoology, etc.). Screening of the remaining titles, abstracts, as well as full-text articles was done by two reviewers (LR and MH) independently, in line with standard procedures used for scoping reviews [13,14]. While we recognise the importance of grey literature (e.g. project reports) in scaling-up processes and initiatives, this literature was not sought for or included in this review (see limitations). Table 1 provides an overview of the final eligibility criteria.

**Table 1:** Inclusion and exclusion criteria. *The eligibility criteria for process evaluations are based on the MRC guidance for the process evaluation of complex health interventions [2].


CRITERIA	SCALE-UP	COMPLEX HEALTH INTERVENTION (CHI)	PROCESS EVALUATION (PE)*

Inclusion	Explicitly state that the aim or objective of the study was related to the **scale-up** of a health care intervention (e.g., integrated care package for Diabetes and Hypertension, exercise-based rehabilitation). **The language used by the study authors was central in assessing this criterion**.	The **intervention** of interest was **complex**. Herein, we follow the description as provided by the UK Medical Research Council, “An intervention might be considered complex because of properties of the intervention itself, such as the number of components involved; the range of behaviours targeted; expertise and skills required by those delivering and receiving the intervention; the number of groups, settings, or levels targeted; or the permitted level of flexibility of the intervention or its components.” [2];	1) The PE entails qualitative and/or quantitative primary research. 2) Only studies that conducted a **PE while scaling up and evaluation of the scale-up process itself** were included. 3) **Explicitly state that a PE was conducted as part of the research study**. The nature of these PEs was the subject of this review, and hence, *a priori* framework or definition was not outlined for these evaluations as such. Therefore, the following criteria were developed. The full text suggested that the study: a) Aimed to conduct a PE in relation to the scale-up of a CHI, or b) conducted implementation research to evaluate structures, resources, and processes in relation to the scale-up of a CHI, or c) evaluated how the scale-up of a CHI produced impact in relation to the scale-up of a CHI, or d) evaluated local context in relation to scale-up of a CHI, or e) a PE was conducted alongside post-evaluation in relation to the scale-up of a CHI.

Exclusion	– Studies not reporting primary research findings (e.g., systematic reviews, editorials, conference proceedings) – Studies in which a PE was conducted to inform *future* scale-up, rather following or along-side a scale-up process.		


The data extraction template was developed iteratively using randomly selected articles. The final extraction template included study demographics (e.g., author, year, country, country’s World Bank income classification [16]), scale-up dimensions (i.e. integration, coverage, comprehensiveness) and strategies [4], and a description of the PE (i.e., quantitative, qualitative or mixed-methods, frameworks adopted, stakeholders involved, and proposed objectives and functions for the PE).

## Results

**Figure 1:**
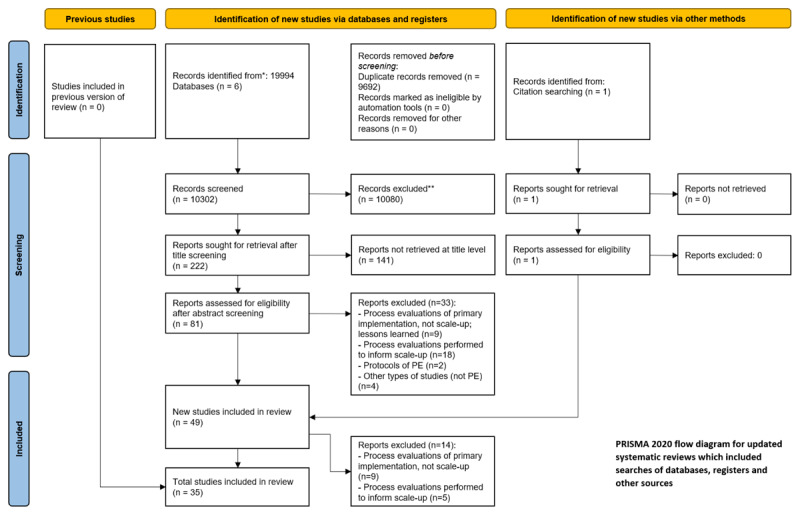
Flow diagram of scoping review in- and exclusion process [79,80] *Databases: PubMed, Embase, CENTRAL, Web of Science, CINAHL, Global Health, Scielo and African Index Medicus; ** excluded manually.

We identified 10,538 unique records (on February 19th, 2024; see Figure 1) of which 56 studies (60 reports) were included after abstract (n = 274) and full-text (n = 133) screening [17,18,19,20,21,22,23,24,25,26,27,28,29,30,31,32,33,34,35,36,37,38,39,40,41,42,43,44,45,46,47,48,49,50,51,52,53,54,55,56,57,58,59,60,61,62,63,64,65,66,67,68,69,70,71,72,73,74,75,76,77,78]. The majority of studies were conducted in high-income countries (n = 23, 41%), followed by upper-middle (n = 11, 20%), low-income (n = 10, 18%) and lower-middle income countries (n = 9, 16%) respectively. Notably, five out of 23 (22%) studies conducted in high-income countries focussed on vulnerable populations (e.g., refugee populations, low socio-economic groups).

### Nature of the CHIs

The CHIs being scaled-up were classified in the following health domains (see Annex 1): communicable diseases (n = 17, 30%), prevention (n = 15, 27%), maternal health and childcare (n = 12, 21%), and non-communicable disease (n = 11, 20%). Two interventions could also be considered at the meso level (e.g., management/governance) [20,27].

A detailed overview of the types of CHIs being scaled-up, and their main dimensions of scale-up (increase coverage, comprehensiveness, institutionalisation) is available in Annex 1. Seven common thematic areas emerged (with some studies covering multiple areas of interest).

- Community health and outreach: home visits by community health workers and community-based distribution (e.g., medication). An example is HPV self-collection through community health workers at home visits [19].
- Chronic disease management: self-management and task-shifting programs. An example is the “Healthy Living for People With Type 2 Diabetes (HeLP-Diabetes)” digital self-management intervention [39].
- Maternal and child health initiatives: home-visit programs, integrated care packages and quality improvement programs. An example is the Happy Child home-visiting program for child development [26].
- Technology and innovation in health care delivery: mobile health interventions and decision support tools. An example is the Extension for Community Healthcare Outcomes (ECHO) model for virtual communities of practice [55].
- Quality improvement is a recurrent thematic area across health domains and care levels. An example is technology-supported decision guides for maternal and child health service delivery [20].
- Prevention and health promotion. An example is the peer-led health promotion program for indigenous populations [28].
- Integrated care or multidisciplinary approaches to address complex health issues more comprehensively or resource efficient. One program integrated PrEP services into routine clinical practice while another integrated mobile health interventions for patients suffering from stroke [35].

### Scale-up strategies

An inductive reflection on reported strategies identified seven key areas to support scale-up of complex health interventions:

- Integration of the CHI into national or local policy
- Capacity building and training, in particular cascade training (i.e. train the trainer) approaches as well as cascade facilitation.
- Resource support (e.g., appointment of staff)
- Quality improvement and monitoring (e.g., benchmarking against quality criteria)
- Cultivating partnerships and collaboration (e.g., peer-support network)
- Transfer of ownership (e.g., shifting external to internal facilitation)
- Ongoing advocacy and communication (e.g., based on impact evaluation)

### Frameworks

Where studies did adopt a framework to evaluate the scale-up process, a wide variety (22 different frameworks from 36 studies) of frameworks were used (see Annex 1). A total of 20 studies did not report an underlying theory or framework. In some instances, frameworks were modified for use in the scale-up process (e.g., choosing different components from different frameworks to fit the objective). The most common frameworks used were RE-AIM (n = 10, 28%), followed by WHO ExpandNet, Consolidated Framework for Implementation Research, Normalization Process Theory, and the Non-Adoption, Abandonment, Scale-up, Spread and Sustainability (NASSS) framework (all n = 2; 6%). Table 2 provides a short description of the most common frameworks and models used and how these frameworks were adapted (if applicable).

**Table 2:** Overview of selected frameworks and theories used to shape the process evaluation during scale-up.


RE-AIM	RE-AIM guides the planning and evaluation of programs according to the five key RE-AIM outcomes: Reach (the target population), Effectiveness (the impact on outcomes), Adoption (the extent to which individuals and settings adopt the intervention), Implementation (the fidelity and consistency of delivery), and Maintenance (the sustainability of the intervention over time) [7]. While mostly used in the evaluation stages, the RE-AIM framework can be used in the implementation of complex interventions by guiding its planning, execution and evaluation. In this review, RE-AIM was mostly used to map different outcomes of interest in relation to the scale-up process.

Framework for Reporting Adaptations and Modifications-Enhanced (FRAME) – Implementation Strategies (FRAME-IS)	FRAME or FRAME-IS is designed to guide researchers and practitioners in reporting adaptations and modifications made to interventions or implementation strategies respectively. It emphasizes transparency and clarity in documenting changes to interventions or strategies, ensuring that the rationale and impact of adaptations are clearly communicated [81]. By reporting adaptations and modifications in the original intervention, using this framework adequately provides information on scale-up.

Integrated Promoting Action on Research Implementation in Health Services (i-PARIHS) framework	i-PARIHS helps understand and guide the implementation of evidence-based practices in health services. It considers the interaction between Innovation (the new practice), Recipients (the individuals and teams implementing the practice), Context (the environment in which implementation occurs), and Facilitation (strategies to support implementation) [82]. This framework promotes that the scale-up is tailored to the specific context.

Normalization Process Theory	This classic theory focuses on understanding how new practices, technologies, or interventions become embedded and integrated into routine work in healthcare settings. It explores the processes through which individuals and groups make sense of, engage with, and sustainably incorporate innovations into their everyday practices [83].

(updated) Consolidated Framework for Implementation Research (CFIR)	CFIR helps identify barriers and facilitators to implementation, guides strategy design, and evaluates implementation outcomes. It looks at five main areas: the intervention itself (its attributes and advantages), the external context (like policies and collaborations), the internal organizational setting (culture, leadership), individual characteristics (knowledge, attitudes), and the implementation process (planning, execution, sustainability) [84].

WHO ExpandNet	ExpandNet is a network and approach developed by the World Health Organization (WHO) to support the scale-up of successful health interventions. Key components include: systematic planning, stakeholder engagement, adaptive management, monitoring and evaluation, documentation and knowledge sharing [85].

Non-Adoption, Abandonment, Scale-up, Spread and Sustainability (NASSS) framework	A conceptual implementation framework developed to understand the complexity of implementing and sustaining health interventions or technologies within healthcare systems. It provides a structured approach for analyzing various factors (innovation, individual, adopting organization, wider context, socio-technical system, implementation process, and outcomes over time) that influence the success or failure of implementing innovations in healthcare settings [86].


### Methods of process evaluation and outcomes

The majority of studies used various forms of qualitative methods (e.g., in-depth interviews, key-informant interviews, focus-groups discussions, observations). Some were supplemented with pragmatic process data from registries, patient records, and progress reports. The time window being evaluated varied between one and four years.

Few studies reported using the process evaluation to adjust scale-up strategies in real-time. While the exact duration of the process being evaluated was often difficult to attain from the report, most evaluations appeared to be retrospective.

Many PEs combined a quantitative approach (e.g., environmental scan, registry data, routine data extraction, observations, training evaluation forms, or surveys) to understand common implementation outcomes (reach, adoption, and fidelity). These quantitative approaches were supplemented by qualitative methods to obtain a better understanding of the mechanisms of scale-up, barriers, facilitators, or additional strategies or recommendations to improve or optimize scale-up. These methods are in line with the reported functions of PEs, being:

– To evaluate structures through which scale-up of CHIs is achieved (84% of studies)
– To evaluate processes through which scale-up of CHIs is achieved (70% of studies)
– To evaluate how external factors (i.e. context) influence the scale-up of CHIs (52% of studies)
– To evaluate how scaling-up affects the CHI at a systems level (i.e. real-world application; 30% of studies)

A wide variety of outcomes for the PE and scale-up process were reported. However, common outcomes of the PE included barriers and facilitators (30%), adoption (including non-adoption and fidelity; 23%), acceptability (12.5%), challenges (11%), and reach (11%).

Stakeholder involvement varied based on the type of intervention, methodology used, and level at which scale-up was being evaluated (e.g., community, facility, and/or national levels). What was notable is that, across levels, the inclusion of end-users in the evaluation process was not clearly defined, even though the intervention targeted the community.

## Discussion

The objective of this review was to describe the current practice of process evaluations associated with the scale-up of complex interventions and associated scale-up strategies. This scoping review, mostly descriptive in nature, aimed to map key trends in the conduct of process evaluations in the context of scaling up complex health interventions, gaps in their underlying methodology or theoretical underpinnings, with the overarching aim to inform targeted research and innovation to strengthen the quality and rigor of such evaluations. Results from this review, drawn from 56 unique studies, consolidates the hypothesis that the field of PEs in the context of scale-up is challenged by heterogeneity and ambiguity in terms of definition, methodology, process, and outcomes thereby underpinning the opportunity to strengthen this field. Herein, we reflect on some of the key findings from this review.

### Key functions of process evaluations within the context of scaling-up

Included PEs focused on evaluation of the context (structures needed (84%) and the influence of external factors on scale-up (52%)), and of the conditions needed to support successful scale-up (70%). When reflecting on the key functions of the process evaluation of CHI, as described by Moore and colleagues [1], there are some clear synergies with process evaluations for scaling-up these interventions. One crucial aspect that emerged is understanding the role of the context where these interventions are being scaled-up to. This becomes especially important when we are trying to expand these interventions to cover larger populations (relative to increasing comprehensiveness or institutionalisation), as different contexts can lead to varying implementation and effectiveness of outcomes. It goes without saying that the strategies to support scale-up can be considered a complex intervention in itself (e.g., train the trainer, cascade facilitation, amongst others). Where current process evaluations may fall short is in the identification of how mechanisms of impact vary in relation to context as well as the identification of unexpected pathways or consequences. While many evaluation frameworks were used to identify factors (e.g., barriers, facilitators) at a specific point in the scaling-up process, not many used repeat evaluations to simulate how these processes unfold over time, how to adapt implementations strategies accordingly, and how to appreciate context plasticity [87]. Systems thinking – an important paradigm promoted by, for instance, the WHO ExpandNet scaling-up framework among others [85,88], allows for untoward consequences and drivers for change to be identified holistically – was reported only in a single study within our review [48].

### Extrapolation of implementation outcomes to support evaluating of scale-up phase

The most common outcomes of the included studies and process evaluations were facilitators and barriers (to scale-up), acceptability of innovation, adoption, fidelity (drift), reach in relation to intended target, and effectiveness (drift). Many of these outcomes are in line with common implementation outcomes (e.g., reach, fidelity) known to various (earlier) stages of implementation. Some outcomes reported, interestingly, would have been expected at earlier phases in the innovation development process (e.g., acceptability). The addition of “drift” (e.g., fidelity drift) to some of the outcomes identified is of specific interest, as more than e.g., fidelity, the notion of “drift” marks the temporal processes involved in scale-up and the more real-world context in which scaling-up is taking place relative to research-driven and controlled experimental or implementation research.

### Strengthening the underlying methodological and theoretical underpinnings

More than 20 different implementation, process or evaluation frameworks were identified, few of which were explicitly developed for the purpose of scale-up evaluation. While we recognise the value of specific implementation frameworks to shape aspects of the scale-up process evaluation, a bespoke framework for the scale-up of complex interventions may aid to support the longitudinal, multi-dimensional, and phased nature of scaling-up complex interventions. The widely used RE-AIM framework was most common (n = 10), and arguably, its domains (e.g., reach) support the transition from implementation to scale-up and is conducive to both quantitative and qualitative methods to support the evaluation. In quite a few cases either bespoke frameworks were constructed through literature or by merging existing features of existing frameworks (e.g., CFIR and ExpandNet), which may reflect different frameworks used for scale-up and process evaluation. Notably, a large proportion of studies (n = 20, 36%) did not report the use of a methodological framework to structure their process evaluation. These findings strengthen our view on the value in expanding the field of research, reporting, and identification of best-practices concerned with scale-up of CHIs.

### Whose views are included?

As alluded to, increasing attention has been placed on the complexity and system dynamics related to scaling-up complex interventions in routine settings – irrespective of the scale-up dimensions [3,88]. As such, it is important that stakeholders included in the process evaluation reflects this complexity. We argue that in the light of complexity and whole-system innovation, both upstream and downstream stakeholders are vital in understanding the scale-up process and outcomes holistically. In this review, we found that inclusion of stakeholders in the process evaluation varied, were not always explicitly mentioned, and that end-users (for example) were often not included. The type of stakeholders involved depended on the type of innovation, dimension(s) of scale up, and operational level of scaling up (e.g., national, community), such that end-users were less likely to be involved once the scale-up process moved further away from the individual, and institutionalization was the primary focus.

### Multi-dimensional and ambivalent process

While increasing coverage of innovations was often the main target, most studies also showed that scale-up implies growth along all dimensions (coverage, comprehensiveness, and institutionalization). Methodologically, this hampered our ambition to map the type of innovation, outcomes, or stakeholders involved relative to the dimensions of scale-up. In other words, seeing certain trends in how process evaluations are conducted in relation to e.g., increasing coverage, or institutionalization. A second methodological challenge was that it was frequently unclear when the primary implementation ended and scale-up starts. This was reflected in factors such as the outcomes reported (e.g., acceptability), the use of methodologies (randomized clinical trials) that were unexpected relative to the scale-up of evidence-based interventions or processes, and difficulties in pinpointing when the process evaluation was conducted relative to the scale-up process.

### Limitations

Despite a robust search strategy and the use of multiple reviewers, this review has several limitations which need to be acknowledged. Most notably, scaling-up from evidence into practice is a process that may take place outside of the academic environment. Hence, grey literature (e.g., funding reports, national programs) — which were not included in this review — may have provided additional insight in terms of the PE’s conducted alongside real-world scale-up processes and their theoretical underpinnings. Conversely, a wide scope of academic literature was searched (i.e., eight data sources), and both backward and forward screening of citations promoted the rigor of the search processes. This wide net, independent of the types of CHIs being studied, provided a comprehensive overview of the current practice (within the academic space) for conducting PEs alongside scale-up, and may increase the generalizability of the findings. Second, there was some ambiguity in terms of the concepts reviewed. While it was generally clear if a project aimed to scale-up an intervention from site one site to another (i.e., increase coverage), the dimensions of integration or comprehensiveness were not always distinct. Similarly, the concept of PE quickly leaned towards qualitative stakeholder engagement while quantitative data can (or should) also support the evaluation of process (e.g., reach across all scale-up sites). The latter may be implicitly reported and not explicitly referred to as process evaluation and therefore not fully captured in this review. The ambiguity as to what constitutes a process evaluation and when do we speak of scale-up reflects, for instance, in the number of citations screened relative to the number of articles included. The nature of a scoping review does allow for the refinement of selection criteria and understanding of these concepts as one becomes more familiar with the literature, and future standardization (e.g., a framework for the PEs alongside scale-up) can resolve some of this ambiguity.

## Conclusion

There is considerable heterogeneity in the current practice of conducting process evaluations alongside (or following) the scale-up of complex health interventions. This heterogeneity may reflect differences in context, conceptual challenges, multi-dimensional nature of scale-up, and the point of engagement with the health care system (e.g., community-level). Ideally, a process evaluation is a recurrent continuous process, levering a systems-driven understanding and triangulation of qualitative and quantitative data, that takes place alongside the scale-up project to inform real-world adaptations to the scale-up strategies and (untoward) mechanisms of impact when applicable.

## Additional Files

The additional files for this article can be found as follows:

10.5334/ijic.7600.s1 Supplementary Material 1. Annex 1.

10.5334/ijic.7600.s2 Supplementary Material 2. Online supplement.

10.5334/ijic.7600.s3 Supplementary Material 3. Supplementary data – Table 1.
