# Supplementary material for: Process Evaluations for the Scale-Up of Complex Interventions – a Scoping Review
Source: Int J Integr Care. 2024 Nov 8;24(4):6. doi: 10.5334/ijic.7600 (PMC11546072; doi:10.5334/ijic.7600)
Supplement: Supplementary Material 1. — Annex 1. [file ijic-24-4-7600-s1.pdf]

Annex 1: Characteristics of included studies about the complex health interventions of, scale-up frameworks, process evaluation methods and outcomes.

**Study characteristics**			**Complex Health Intervention**			**Scale-up**			**Process evaluation**		
**Studies**<br><br>**(n = 56)**	**Country**	**Country('s) income classification ****	**Description of CHI**	**Target population for CHI**	**Health problem targeted[$]**	**Scale-up dimensions (P, I, C)^**	**Framework used**	**Key scale-up strategies identified**	**Process evaluation methods**	**Process evaluation sample / sources**	**Reported process evaluation outcomes**
Ajayi et al 2008 [17]	Nigeria	LM	Artemisinin-based combination therapy in the context of home management of Malaria	Forty rural communities within the Onaara government area	CD	I, C	n/a	- Adoption into national policy<br><br>- Training to build capacity (community medicine distributors)<br><br>- Financial incentives	Focus-group discussions (n = 10) within randomly stratified selected community representatives, supplemented by key informant interviews	Three groups of young mothers and old mothers each, and two of young fathers and old fathers each, were involved in the FGD while three patient medicine sellers, four opinion/ community leaders, and two health workers were interviewed during the key informant interviews.	- Feasibility<br><br>- Accessibility<br><br>- Safety
Allen et al. 2023 [18]	Australia	HIC	EDDIE+: education and training of all nursing and personal care workers (PCWs); use of decision support tools; use of diagnostic equipment for clinical assessment and monitoring; and facilitation and clinical support	Elderly	Prevention	P, C	Integrated Promoting Action on Research Implementation in Health Services (i-PARIHS) framework	- Implementation toolkit and refinements to intervention and decision support tool<br><br>- Training and facilitation (intervention specific)<br><br>- Resource support (e.g., diagnostic equipment)	Environmental scan (outer context), organizational and local RAC home context assessments, intervention working group	A review of relevant programs and policies nationally, and evidence relating to hospital avoidance and the aged care sector; The organizational level context assessment was carried out virtually by the nurse educator (EDDIE + study team)<br><br>and key clinical executives from the aged care provider; The local context assessments were conducted in 11 RAC homes.	- Identify needs for intervention refinements<br><br>- Barriers and facilitators to implementation<br><br>- Strategies to overcome barriers (e.g., decision support tool)
Arrossi et al 2017 [19]	Argentina	HIC	Human papillomavirus self-collection through community health workers at home visits	Women aged 30+ attending public health services without a cervical screening test	Prevention, NCD	P, I	RE-AIM and adaptation of health system framework	- Training of community health workers<br><br>- Quality	Scale-up was nested within the Jujuy demonstration	Thee linked data sources were used: Public Health Care census (2013), the SITAM (national	- Drivers for scale-up<br><br>- Effectiveness

				in the past 5 years				improvement refreshment training	project.<br>- Analysis of the scaling-up process using an adaptation of the Health System Framework (HSF); four domains (stewardship, organizational capacity, technology/strateg y, Funding). Analysis based on content analysis of program reports and observation derived from participation in policy definition.<br>- Analysis of dimensions proposed by RE-AIM through quantitative analysis using routine data sources	information system), and a self-administered questionnaire requested from all involved CHWs.	retention<br>- Acceptance<br>- Adoption<br>- Implementation barriers
Bennett et al 2017 [20]	Afghanistan , Bangladesh, Uganda	LIC, LIC, LM	Three case-examples of quality improvement interventions (community scorecard, technology supported decision guides, and package of interventions to support maternal health services)	Rural poor	MCH, NCD, Manageme nt	P, I, C	n/a	- Adoption into policy (Afghanistan)<br>- Quality improvement collaboration and coordination to facilitate institutionalization	Four dimensions of scale-up conceptualized: attributes of the innovation or service, attributes of the target community and actors involved in scale-up, context, and scaling-up strategy. Drawing primarily on project documentation the author sought to describe these four dimensions for the three study	Project documentation including research proposals, theories of change, country presentations at annual meetings, routine monitoring reports, annual reports, country publications including articles and policy briefs. Triangulation was supported by sharing the analysis with the country teams for input.	- Attributes of the innovation or service (appropriateness, fidelity)<br>- Attributes of the target community and actors involved (acceptability, intention to adopt, coverage)<br>- Context (feasibility)<br>- Scaling-up strategies

									contexts		(implementation cost, sustainability)
Benton et al. 2022 [21]	United Kingdom	HIC	Healthy living; A digital self-management intervention that has demonstrated effectiveness is Healthy Living for People With Type 2 Diabetes (HeLP-Diabetes), mainly consisting of a theory- and evidence-based website.	Patients with Type 2 Diabetes	NCD	P, I, C	n/a	- Adoption of intervention as National Health Service as part of routine care<br><br>- Technical assistance (externally commissioned)	Content analysis of healthy living service (i.e. website in beta version) available. Qualitative interviews	Stakeholders with high-level involvement and with a variety of roles in HeLP diabetes or Healthy Living (e.g., academic researchers, content developers, program managers)	- Fidelity<br><br>- Fidelity drift (reasons for)<br><br>- Describe content and features of intervention delivery following national roll-out
Bottorff et al 2021 [22]	Canada	HIC	Healthy Together program designed to engage families in an interactive program to support healthy lifestyles	Caregivers and their children (up to 18 years of age)	Prevention	P, I	RE-AIM framework	- Training of facilitators<br><br>- Support knowledge mobilization framework<br><br>- Support policy changes	External university-led evaluation based on RE-AIM framework using consolidated participant surveys for children, youth, caregivers and facilitators as well as observation checklist to assess implementation fidelity.<br><br>Interviews were held with directors of participating organizations and members of the national advisory committee.<br><br>Social return on investment analysis	Program participants, program facilitators, national advisory committee.	- Reach<br><br>- Adoption / changes in program delivery<br><br>- Implementation<br><br>- Effectiveness<br><br>- Maintenance<br><br>- Identify new opportunities to extend impact<br><br>- Develop strategies
Brien et al. 2023 [23]	India	LM	Enabling Inclusion app to support rehabilitation specialists and community rehabilitation workers to screen, assess, and	Children with disabilities	MCH	P, C	Monitoring, evaluation, and learning (MEL) system based on the '5 aspirations of	- Cultivation of partnerships with policy makers<br><br>- Licensing app to other NGOs	Embedded program indications, including program staff / stakeholder use, demographic and socio-	Program staff / stakeholders, children with disabilities and caregivers, NGO program managers, funders, service	- Evaluation of an implementation strategy (Monitoring, Evaluation and Learning system) on scale-up

			monitor progress of children with developmental disabilities, especially in rural areas, and to provide child rehab service providers (e.g., therapists, community rehab workers, NGO management, and caretakers) with a menu of evidence-based, family-centered interventions.				Measurement for Change	- Form knowledge partnerships with government institutions	economic indicators of end-users, children development screening information, access to care services, and others); key performance indicators per program site (e.g., type of impairment, program adherence rates); qualitative and quantitative methods feedback from NGO program managers, funders, service providers, beneficiaries, public sector policymakers, and the community	providers, policy makers and broader community.	(reach).
Bright et al. 2023 [24]	Sierra Leone	LIC	Implementation of Community Blood Pressure monitoring in Rural Africa: Detection of underLying pre-Eclampsia (CRADLE) vital signs alert device with a training package for healthcare professionals	Women (at risk of) Pre-eclampsia	MCH	C	NASSS (non-adoption, abandonment, scale-up, spread, sustainability)<br><br>Framework	- Train the trainers model<br><br>- Provision of implementation materials	An existing database of training registers, held by the implementing organization, was used to calculate the proportion of facilities trained per district; Focus Group Discussions were held. Analysis was primarily deductive, using the domains of the NASSS Framework as a priori themes	Champions in the four selected districts,	- Identify inter-district variation in adoption<br><br>- Factors impacting adoption<br><br>- Provide guidance to avoid non-adoption and abandonment
Brunton et al 2022 [25]	United Kingdom	HIC	ABC care bundle for patients with stroke, including rapid anticoagulant reversal,	Patients with Stroke	NCD	I, C	i-PARIHS	- External facilitation team<br><br>- Set-up of internal quality	Semi-structured interviews, non-participant observation at	External facilitators, internal project teams, and clinicians; seventy-	- Prospective changes in implementation<br><br>- Clinicians’

			delivery of intensive blood pressure lowering, adherence to care pathway including immediate neurosurgical referral where applicable.					improvement teams<br><br>- Quality improvement workshops<br><br>- Development of decision support app and dashboard	meetings / events and analysis of relevant project documents	nine hours of non-participant observation of quality improvement workshops, collaborative meetings, and local site meetings.	experience with ABC care bundle<br><br>- How context influenced implementation
Buccini et al. 2024 [26]	Brazil	UM	PCF (Happy Child Program in Portuguese); home-visiting program for child development	Early childhood development	Prevention, MCH	P, I, C	RE-AIM	- National adoption and roll-out of intervention<br><br>- Operationalization of implementation at municipal level<br><br>- Technical support at provincial and national level<br><br>- The above was achieved through acquisition and training of human resources, supervision and monitoring, home visits, initiation of complementary actions, and acquisition of funding.	Comparative case study analysis in contrasting municipalities; in-depth interviews	Municipal managers, supervisors, home visitors, families and cross-sectoral professionals (n = 244)	- Identification of barriers and facilitators<br><br>- Achieving implementation outcomes (based on RE-AIM)
Bulthuis et al. 2023 [27]	Ghana, Malawi, Uganda	LM/LIC/LIC	To reinforce management capacities of District Health Management Teams (DHMTs) (management strengthening intervention) through an action-research cycle	n/a	Manageme nt	I, C	ExpandNet	- Establishment of national steering group and resource team	Comparative multi-national case study including interviews and focus group discussions.	Interviews and group discussions took place with district, regional and national stakeholders who were involved in the implementation and scale-up of the<br><br>intervention.	- Shared vision amongst stakeholders about facilitators that support institutionalize intervention into existing systems
Cama et al. 2024 [28]	Australia	HIC	Peer-led health promotion program that aimed to improve access to screening and treatment for blood	Aboriginals and Torres Strait Islanders	Prevention, CD	P, I, C	RE-AIM	Unclear	Semi-structured interviews	Four Aboriginal and Torres Strait Islander and 11 non-Aboriginal or Torres Strait Islander health	- Successes<br><br>- Challenges<br><br>- Real-world

			borne viruses and sexually transmissible infections in indigenous populations.							workers, as well as 33 Aboriginal and Torres Strait Islander clients	implementation (using RE-AIM)
Cintyamena et al 2021 [29]	Indonesia	UM	Polio immunization		CD	P, I, C	WHO ExpandNet; CFIR	- Planning; develop scaling up target output and innovation<br>- Identifying user organization and resource team<br>- Analysing environment<br>- Deciding and implementing strategic<br>planning<br>- Monitoring and evaluation	Document review (grey literature), survey questionnaire, and qualitative analysis were conducted to describe context and the polio program timeline.	Key informant interviews with main actors on polio eradication program at both national and sub-national level. Survey responses from 323 program participants (>1 year exposure)	- Program challenges<br>- Strategies<br>- Lessons learned
Dolan et al. 2023 [30]	Kenya	LM	Table-based Electronic Immunization Registry (EIR) with clinical decision support features.	Childhood immunization	Prevention, CD	C	Expanded usability heuristics based on Miller et al. 2018	- Cascade training approach<br>- Development of a WhatsApp based peer-support network	Qualitative data through structured interviews at 12 purposefully selected user sites and direct workflow observations	Unclear ("users")	- Mechanisms of integration<br>- Development of refined program theory
Fikree et al 2020 [31]	Ethiopia	LIC	Long-acting reversible contraception – implants and intrauterine devices – in youth friendly one-stop-shop services	Youth at family planning unit	Prevention, CD	P, I	Bespoke analytic framework drawn from various sources comprising<br>- Stakeholder engagement<br>- Roles and responsibilitie s<br>- Policy environment	- Training of Youth Friendly service providers<br>- Training of peer-educators to debunk myths about services	Face-to face semi-structured key informant interviews and data extraction from the Health Management Information System family planning registers	Senior managers (n = 25) and technical staff (n = 31) from 2 sources (public sector and implementing partners).<br>Data extraction from public sector family planning registers maintained at eight health centres	- Facilitating factors<br>- Challenges<br>- Barriers

							- Financial resources<br><br>- Quality of voluntary family planning services<br><br>- Data availability and use				
Fischer et al 2019 [32]	South Africa	UM	NurseConnect; a platform based on the Integrated Behavioural Model and Adult Learning Theory of change aimed to support nurses and midwives in maternal and childcare	Nurses and midwives in maternal and childcare	MCH	P, I	n/a	- Quality improvement collaboration	Focus group discussions or in-depth interviews at 18 randomly selected facilities.	Nurses and midwives (n = 110) registered on NurseConnect	- To optimize user experience<br><br>- To inform expansion of NurseConnect
Georgeu et al 2012 [33]	South Africa	UM	Nurse-initiated and managed antiretroviral treatment; streamlining tasks and roles to expand treatment and care for HIV (STRETCH)	Patients with HIV/AIDS	CD	P, I, C	Assessment of the fidelity of the implementation of the intervention under study	- Educational outreach training/ train the trainer to train model [34]	In-depth interviews, focus group discussions, data observations	Patients, health workers, health managers, and other key informants	- Perceptions of task-shifting model<br><br>- Changes in health provider roles<br><br>- Attitudes and patient relationships<br><br>- Impact of context on implementation outcomes
Gong et al 2021 [35]	China	UM	Primary-care-based integrated mobile health intervention (SINEMA) for patients with Stroke	Patients with Stroke	NCD	P, I, C	RE-AIM / MRC process evaluation framework<br><br>The RE-AIM framework was used to inform the measurement of implementatio	- Training of village doctors<br><br>- Performance-based financial support<br><br>- Virtual-group peer support<br><br>- Development of digital infrastructure to support program	Process evaluation alongside a type 2 hybrid implementation trial in 50 rural villages<br><br>Multiple data collection approaches were adopted, including self-administered	Stakeholders (qualitative), village doctors (quantitative). Stakeholders included patients who suffered stroke, village doctors, physicians at township hospitals, and a<br><br>county coordinator	- Implementation indicators<br><br>- Enablers<br><br>- Barriers<br><br>- Potential impact pathways that affect effectiveness

							n outcomes, covering program reach, adoption, implementation, and maintenance.<br><br>The MRC process evaluation framework was used to investigate the interactive relationship between context, intervention components, and implementation indicators.	delivery	surveys among village doctors, face-to-face interview-based surveys among participants, monitoring data from the digital health system, and in-depth interviews among stakeholders		
Hargreaves et al 2010 [36]	South Africa	UM	Intervention with Microfinance for AIDS and Gender Equity (IMAGE). Multi-component intervention including poverty-focused microfinancing facilitated by a fieldworker, gender and HIV/AIDS awareness training, and community mobilization.	Patients with HIV / AIDS	CD	P	n/a	- Change in delivery model from "linked" to "parallel"<br><br>- Policy alignment<br><br>- Activities to promote collaboration<br><br>- Staff recruitment and training (by existing staff)	Semi-structured interviews.<br><br>Finance monitoring systems provided data on loan repayment	Manager, field staff, trial client, and scale-up client interviews.	- Feasibility<br><br>- Acceptability
Hollis et al. 2022 [37]	Australia	HIC	Train-the-trainer model for scaling-up a "health conversations skills (HCS) training"; HCS training uses an interactive, participatory approach to learning built on the pedagogy of active learning, with the trainer modelling the	Health professionals engaged in behaviour change conversations	Prevention	P	Taxonomy of Behaviour Change Techniques / Theoretical Domains Framework	- Train the trainer	Pre-post training surveys	Health conversation skills trainees	- Perceived barriers and facilitators in delivering the intervention based on the Theoretical Domains Framework.

			HCS philosophy and skills throughout.								
Holroyd-Leduc et al 2019 [38]	Canada	HIC	Mobilization of Vulnerable Elders (MOVE) intervention; an interprofessional approach that focuses on early and consistent mobilization of older adults admitted to hospital.	Elderly admitted to hospital	R	P, I, C	Select, Tailor, Plan and Engage (STEP) tool based on theoretical domains framework	- Initiation of site-implementation teams<br>- Benchmarking / quality improvement through a bespoke online tool for monitoring and evaluation<br>- Implementations support tool based on sustainability findings	Completion of NHS sustainability survey	Four participating units	- Changes in the climate of sustainability<br>- Identify additional sustainability considerations
Hunt et al 2020 [39]	United Kingdom	HIC	Gender-Sensitized Weight Management and Healthy Living Program	Overweight men	Prevention	P, I, C	PRACTical planning for Implementation and Scale-up (PRACTIS)	- Easy-access license shifting to single license model<br>- Transfer ownership<br>- Training program to train new coaches / clubs (train the trainers package)<br>- Creating a support structure to enable quality assurance	Comparison of baseline data between post-RCT participant metrics (socio-demographic, weight, waist circumference, blood pressure) and scaled-up routine program participant metrics.<br>Mapping of scale-out adaptations to common scale-out typology	Data from 42 Scottish professional football league participants.	- Reach<br>- Retention of effectiveness<br>- Scale-out adaptations
Hyam et al. 2024 [40]	United Kingdom	HIC	A trans-diagnostic service model and care package for young people aged 16–25 years with a recent onset eating disorder that is 3 years or less in duration. FREED encourages rapid access to treatment that	Patients with eating disorders	Mental health	P, C	RE-AIM and Non-adoption, Abandonment, and Challenges to Scale-Up, Spread and Sustainability (NASSS) framework	- Integration in national adoption and spread program<br>- Regional implementation specialist' support	Individual structured interviews according NASSS complexity assessment tool	National program leads	- Perceived sustainability of the model

			is well-coordinated, evidence-based, and adapted to young people's needs								
Irungu et al 2021 [41]	Kenya	LM	Integration of PrEP services into routine clinical practice.	Patients with HIV / AIDS at public HIV clinics	CD	I, C	The expanded framework for reporting adaptations and modifications (FRAME)	- Training of health care providers through project staff<br><br>- Technical assistance and mentorship<br><br>- Sharing of best-practices<br><br>- Summary reports benchmarking service delivery one-year post initiation	Semi-structured interviews	Health care providers at selected public HIV clinics, including clinical officers, nurses, social workers, and counsellors.	- Identify real-world mechanisms of implementation<br><br>- Adaptations to context to support implementation
Kauchali et al 2022 [42]	South Africa, Bolivia, Malawi, and Ghana	UM, LM, LIC, LM	Four quality improvement cases based on WHO guidelines for severe acute malnutrition.	Children with severe acute malnutrition	MCH	P, I, C	n/a	- Participatory approach to capacity building<br><br>- Policy adaptation (local and national)<br><br>- Evaluation of data for action and advocacy<br><br>- Train the trainer (champions)<br><br>- High-level team to link intervention to insurance system	Contemporaneous implementation reports, qualitative and quantitative data from our research papers, and our prospective and retrospective data collection	Four country studies of scaling up implementation of WHO guidelines for improving the inpatient management of severe acute malnutrition within under- resourced public sector health services	- Identification of key factors informing successful scale-up<br><br>- Lessons learned
Kenis et al. 2023 [43]	Belgium	HIC	Self-directed co-creation of a care pathway for patients treated with oral anticancer drugs.	Patients with Cancer	NCD	I, C	n/a	- Shifting ownership / self-directed approach<br><br>- External coaching at group level<br><br>- Supportive tools<br><br>- Creation of a learning	Semi-structured interviews	Project coordinators, project members,	- Influencing factors for successful scale-up

								community		
Kinney et al. 2023 [44]	South Africa	UM	Perinatal audit program		MCH	I, C	Health policy analysis triangle framework	- Guidelines for institutionalization<br>- Incentives for ongoing professional development<br>- Intentional and continuous demonstration of impact<br>- Continuous implementation support<br>- Assembly of a national committee	56 individual interviews, a systematic document review, administration of a semi-structured questionnaire, and 10 nonparticipant observations of meetings related to the perinatal audit program	Key informants at different health system levels (e.g., national, provincial, regional, subdistrict, facility, primary health care) and stratified by subdistrict (n = 5)	- Historical mapping / lessons learned
Lafferty et al 2021 [45]	Australia	HIC	The Test Treat ANd GO (TTANGO) trial; sexually transmissible infections point-of-care testing in remote communities.	Remote / rural indigenous populations	CD	P, I, C	Increasing the scale of population health interventions: A Guide / Acceptability Framework.	Unclear	Semi-structured interviews	15 healthcare workers (including nurses and Aboriginal health practitioners) and five managers (including clinic coordinators and practice managers).	- Acceptability<br>- Barriers to integration<br>- Enablers
Liu et al 2020 [46]	Canada	HIC	10-week MEND behaviour change program focusing on increasing physical activity, reducing sedentary behaviour and promoting healthy eating using practical and engaging activities to deliver information	Children and adolescents aged 7 – 13 with a BMI $\geq 85^{th}$ percentile	MCH	P	RE-AIM	- Capacity building	Stakeholder interviews, leader feedback surveys, document review, and participant feedback surveys. This process evaluation was embedded with a Type 3 hybrid implementation trial.	Stakeholders, participants, parents, program staff across 27 sites	- Reach<br>- Attendance<br>- Fidelity<br>- Acceptability<br>- Satisfaction<br>- Facilitators / Challenges to implementation<br>- Effectiveness
Low et al 2021 [47]	Malaysia	UM	Enhanced Primary Health Care (EnPHC) Initiative including the deployment of triage counters, introduction	Twenty primary health care clinics across two states in Malaysia	NCD	P, I, C	n/a	- Infrastructure development (triage counters, introduction of care coordinator	Structured observation, self-reported assessment form, and in-depth	Clinic liaison officers at the 20 primary health care clinics; Interviews conducted with 20	- Fidelity<br>- Feasibility

			of care coordinator role, NCD screening, NCD risk management, and referral systems					role)	interviews.	health care professionals purposefully selected across different staff categories,	- Adaptation<br>- Benefit
MacGregor et al 2018 [48]	South Africa	UM	Antiretroviral Therapy adherence clubs	Patients with HIV / AIDS	CD	P, I, C	Complex adaptive system lens underlying process evaluation	- Acquisition of funding<br>- Institution of model as policy by provincial government<br>- Recognition of impact<br>- Advocacy on impact	Observations, interviews, evaluation of key adherence club documents, and routine monitoring data	Patient, nurses, doctors, HIV counsellors, pharmacist and pharmacy assistant, data clerk, facility managers and ARV program heads, club coordinator, NGO representatives, government stakeholders (total n = 45)	- Planned organizational change through intervention in a state health system<br>- describing and analysing the core elements necessary for rolling out the club model to new clinics, and for scaling up the number of clubs within a particular facility<br>- Challenges and enablers in bringing the innovation to scale
Marsh et al. 2014 [49]	Ethiopia	LIC	Integrated community-based case management	Non-disease specific	unclear	C	n/a	- Quality of care benchmarking evaluation	Consensus-based evaluation of 70 different benchmarks against observations	Six national experts currently engaged in documenting Ethiopia's iCCM experience and an observer. Experts included two Health Specialists from UNICEF, two advisors from John Snow International's Last 10 Kilometres (L10K) Project, a Save the Children Senior Newborn Health Advisor seconded to the Federal Ministry of Health, and an advisor to the United States Agency for International	- Integrated case management performance against benchmarks across different phases of implementation (including scale-up)

										Development's Integrated Family Health Program. The newly joined Child Health Team Leader of the Federal Ministry of Health was the observer.	
Mary et al 2019 [50]	Niger	LIC	Three interventions to reduce post-partum hemorrhage: misoprostol, uterine balloon tamponade, non-pneumatic anti-shock garment.	Pregnant women	MCH	P, I, C	n/a	- Cascade training<br>- Nationwide procurement	Mixed methods approach including cross-sectional health facility assessments, including provider interviews, and household surveys	A total of 69 health facilities: two national tertiary hospitals; seven regional tertiary hospitals; 12 district secondary hospitals; and 48 primary health centres	- Evaluate program implementation (rates)<br>- Evaluate training and capacity to provide post-partum haemorrhage according to guidelines (i.e. fidelity)
McInnes et al. 2024 [51]	Australia	HIC	Nurse-led, evidence-based protocols to manage fever, hyperglycemia and swallowing protocols following stroke through cascade facilitation model.	Stroke	NCD	P	n/a	- Cascade facilitation	Qualitative process evaluation	Executive / Steering committee members, Angel team leaders, angel consultants, country coordinators.	- Factors that influenced stakeholder engagement during scale-up<br>- Barriers and facilitators that affect implementation<br>- Participants' role in the project / scale-up
McLaughlin 2021 [52]	Australia	HIC	Physical Activity 4 Everyone (PA4E1) seven school physical activity practices	School children	Prevention	P, I, C	Framework for Reporting Adaptations and Modifications -Enhanced (FRAME)	- Package of seven implementation support strategies including 23 sub-strategies [53]	A list of adaptations was generated from a comparison of the efficacy and scale-up trials via published PA4E1 protocols, trial registrations and information provided by trial investigators. Monthly trial team meetings tracked	n/a	- Modifications during scale-up (extend, types, reasons)

									and coded modifications in "real-time" during the conduct of the scale-up trial. The extent, type and reasons for both adaptations and modifications were summarized descriptively		
Morgan et al 2022 [54]	Canada	HIC	Rural primary care-based memory clinics	People with dementia	NCD R	P, I, C	n/a	- Feedback on impact through research data<br>- Dissemination of project findings as advocacy tool (supporting professional academic development)<br>- Clinical support to teams<br>- Regular working group and check-in meetings<br>- Matching of new members with mentors<br>- Ongoing education sessions	Focus-group interviews (n = 6; 40 participants), workgroup meetings (n = 15; 100 participants)	Health care providers involved in the design and delivery of the memory clinics, including family physician, occupation therapist, nurses, social work, physiotherapist, dietician, care coordinator, clinic facilitator, manager, office staff.	- Perspectives of factors influencing sustainability<br>and scale-up
Mubanga et al. 2023 [55]	Zambia	LM	The tele-mentoring Extension for Community Healthcare Outcomes (ECHO); The ECHO model uses videoconference technology to create virtual communities of practice that allow providers to care for patients with complicated health problems, which they would otherwise refer to a higher level of	Patients with HIV and/or TB	CD	P	n/a	- Train the trainer model ("each expert contributes to the development of 20 health care workers)<br>- Community of practice	Health worker survey, focus group discussion, evaluation of routine monitoring and reporting systems for key performance indicators	Interviews with HIV / TB patients (n = 8* 12); survey of 88 health care workers.	- Elicit common experiences<br>- Emergent patterns at impact, knowledge,<br>practice level, as well as factors enabling and hindering ECHO implementation in each province

			care.								
Mulat et al 2022 [56]	Ethiopia	LIC	Community-based health insurance scheme	Public	Prevention	P, I	n/a	- Expand eligibility as part of national, government-driven, scale-up	Semi-structured interviews	Eighteen stakeholders working on health financing and health insurance	- Success factors (or good practices) to scale up<br>- Major challenges to scale-up
Napierala et al. 2019 [57]	Zimbabwe	LM	HIV self-testing program through distribution in outreach sites / clinics	HIV / AIDS	CD	P	n/a	- Peer educators<br>- Co-location within government facilities<br>- Discreet locations<br>- Staff – patient engagement training	Analysis of routine program data, focus group discussions	Female sex workers, peer-educators, and service providers	- Identify additional approaches to HIV self-testing<br>- Identify reasons for non-self-testing / access to care
Naughton et al. / Sharma et al, / Wamuti et al. 2023 [58–60]	Kenya	LM	Assisted partner service (notification for sexual partners of people diagnosed with HIV)	Patients with HIV / AIDS and sexual partners	CD	P, I	Theoretical framework of acceptability	- Training<br>- Provision of manuals<br>- Monitoring and evaluation	Phone-based in-depth interviews	Female patients with HIV and male sexual partners	- Acceptability
Niang et al. 2023 [61]	Burkina Faso	LIC	Seasonal malaria chemoprevention (SMC)	Malaria	CD	P, I, C	An integrative conceptual framework of scale-up and sustainability of social innovations in global health	- Cascade facilitation<br>- Support from technical and financial partners<br>- Follow-up and supervision across various hierarchical levels<br>- Using human resources from various health systems<br>- Logistical support, training, communication, monitoring tools	Maximum variation sampling to obtain the broadest range of perspectives for individual interviews; in addition, documentary analysis and observation	Stakeholders at different scales of innovation implementation at national, regional or location organizations.	- Interconnectedness processes underpinning<br>Innovation, sustainability and scaling up in time and space.

								- Create synergy between different actors			
Nsangi et al 2019 [62]	Uganda	LIC	Informed health choices primary school resources aimed at teaching children how to assess trustworthiness of claims about the effects of treatment.	Primary school children	Prevention	P, I	Bespoke framework based on review of existing frameworks and studies of barriers and facilitators to implementing change at schools.	Unclear	Qualitative data using lesson evaluation forms, observation, individual interviews and focus group interviews	Lesson evaluation forms and classroom observations across 60 primary schools. Interviews with district education officers, head teachers, teachers, and children.	- Identify factors affecting the implementation, impact and scaling up of the intervention<br>- Identify potential adverse and beneficial effects of the intervention
Oladeji et al. 2023 [63]	Nigeria	LM	Training of trainers to support the scale-up of WHO Mental Health Gap Action Program Intervention Guide	Perinatal depression	Mental Health, MCH	P, C	Replication Effective Framework (REP)	- Development of an intervention guide<br>- Train the trainers	The training sessions facilitated by these trainers were observed and rated for fidelity by specialist trainers, while the trainees provided their impression of and satisfaction with the training sessions using predesigned assessment forms.	Participants of refresher trainings 7 months post initial cascade training initiation.	- Experiences of provider with delivering care<br>- Identify areas of deficiencies<br>- Retention in knowledge and attitudes
Pichon et al 2021 [64]	USA	HIC	Community-based, accessible, PrEP navigation services; client navigators provide individualized support for clients to access medical and social services, promote reengagement in care, maintain medication adherence, and keep track of medical appointments	Patients with HIV / AIDS	CD	P, I	n/a	- Integration into national HIV/AIDS strategy and regional strategy<br>- Employment of PrEP navigators<br>- Training<br>- Ongoing technical assistance	In-depth qualitative interviews	In 2018, nine PrEP navigators were interviewed; in 2019, five of the original navigators were interviewed again.	- To determine facilitators and barriers to the implementation of the<br>Memphis-based Project PrIDE PrEP navigation activities.<br>- Define best practices
Puchalski Ritchie et al 2021 [65]	Malawi	LIC	Implementation strategy (cascade training) to provide lay health workers with the knowledge, skills and tools needed to	Patients with Tuberculosis	CD	P, I	RE-AIM	- Educational outreach through peer educators<br>- Peer support network	Interviews; document review of training logs, quarterly meeting notes, field visit reports, and study	Lay health workers, peer-trainers, and patients/guardians	- Reach<br>- Adoption<br>- Implementation

			implement an intervention to support patient tuberculosis (TB) treatment adherence					- Clinical support tool	team member meeting notes		- Maintenance<br><br>- Barriers and facilitators to implementation, scalability and sustainability.<br><br>- Suggestions for program improvement
Redwood et al. 2023 [66]	United Kingdom	HIC	Intervention to protect preterm infants from neurodevelopmental disabilities.	Preterm infants at risk of neurodevelopmental disabilities.	MCH	I, C	Normalization Process Theory	- Quality improvement toolkit<br><br>- Implementation guide<br><br>- Regional training<br><br>- Regional support<br><br>- Local champions with funded dedicated time<br><br>- Learning and celebration events<br><br>- Collateral funding	Interviews conducted with key individuals in leadership positions across 150 maternity units	Those providing quality improvement related leadership and regional clinical leads providing clinical leadership to units in their region.	- How different implementation contexts and perinatal team dynamics — frequently lost<br><br>in accounts of barriers and facilitators — generated the observed implementation outcomes.
Renju et al 2010-11 [67–69]	Tanzania	LIC	School-based, teacher-led, peers-supported, youth-friendly services for adolescent sexual and reproductive health intervention scaled-up through training cascade.	School-based adolescents at risk for sexual and reproductive health challenges	Prevention	P, I, C	n/a	- Training cascade from regional through district and ward level to the schools<br><br>- Training guides	Observation of teacher training sessions (coverage, attendance, selection, motivation, experiences, attitudes, perceptions, characteristics, ownership, training content and delivery, levels of support, logistics and other external factors); self-administered questionnaires; interviews, focus-group discussions,	Academic teachers, District trainers and supervisors, guardian teacher, head teacher, regional trainers and supervisors, ward education coordinator.	- Facilitators<br><br>- Inhibitors<br><br>- Teacher attitudes and experiences

									unstructured interviews		
Rhodes et al 2020 [70]	USA	HIC	Evidence-based community-<br><br>level intervention designed to increase HIV and STI prevention behaviours among Spanish-speaking.	Heterosexual Latinx men who are members of recreational soccer teams in the US.	Prevention	I	Scale-up and Spread framework	- Training of navigators	Archival data and individual / small-group interviews. Archival data included partnership meeting notes, progress reports, conference presentations and biweekly meeting notes	Project coordinator supervisors, executive directors, financial managers, development directors, and board members at two time points.	- To examine the implementation process mapped on the 12 Scale-up and Spread constructs.
Sako et al 2018 [71]	Ethiopia	LIC	Community-based grain bank intervention;	Children at risk of severe malnutrition	MCH	P, I, C	A framework describing six elements of successful scaling up: socio-political context, attributes of the intervention, attributes of the implementers, appropriate delivery strategy, the adopting community, and use of research to inform the scale-up process	- Establishment of performance indicators<br><br>- Integration in existing structures<br><br>- Cascade training	Key informant interviews and 31 focus group discussions with purposely selected informants across 8 (out of 180) localities)	Government officials at national, regional and local level, UNICEF, NGO representatives, University stakeholders, Agricultural sector, health sector, and community members.	- Enablers<br><br>- Barriers
Sivakumar et al 2022 [72]	Canada	HIC	eHealth intervention to individualize diabetes care priorities for patients through interactive goal setting, as well as to support an interprofessional team in navigating the complex guideline recommendations of diabetes through shared decision	Patients with Diabetes	NCD	P, I	Normalization Process Theory	Unclear	Individual end-user interviews and a single focus-group discussion. Thematic analysis framework applied to identify construct interactions	End-user participants; focus group discussion within ministry of health.	- Factors critical to scale-up<br><br>- Adaptability<br><br>- Compatibility with similar innovations<br><br>- Consistency with policy directives

			making.								- Cost-effectiveness and affordability
Spaaij et al. 2023 [73]	Switzerland	HIC	Problem Management Plus (PM+) - an evidence-based low-intensity psychological intervention delivered by non-specialist "helpers"	Refugees	Mental health	P, I, C	n/a	Unclear	Semi-structured interviews	Syrian refugees who previously participated in intervention, intervention helpers, health professionals working with refugees, and decision makers from migration integration, social and health sectors.	- Factors influencing the process of larger scale implementation<br><br>- Develop recommendations to guide implementation process.
Tonnon et al 2016 [74]	Netherlands	HIC	Lifestyle intervention (Health Under Construction; HUC); The HUC intervention promotes behavioural change regarding physical activity and a healthy diet through Individual counselling. Employees are screened by an occupational physician for their cardiovascular disease risk during a periodic medical examination	Construction workers (at risk)	Prevention	P, I	n/a	- Contracting commercial health services	Quantitative data using registration sheets, self-report questionnaires, audio-recordings of consultations made by counsellors.	Counsellors, participants / construction workers	- Components reached<br><br>- Dose received<br><br>- Dose delivered<br><br>- Counsellor competence<br><br>- Satisfaction of counsellors and participants with the intervention<br><br>- Barriers experienced by counsellors
Verhey et al. 2022 [75]	Zimbabwe	LM	Friendship Bench (FB) is a brief psychological intervention delivered by Community Health Workers - CHWs which has shown effectiveness for predominantly female primary health care clinic users	Patients attending primary care clinics	Prevention, Mental health	P, I, C	RE-AIM	- Formal hand-over to city health department<br><br>- Development of data collection tools to integrate into routine primary health care services<br><br>- Sustain technical supporting role	Review of intervention data / activities at 36 primary health clinics (not reliably available); questionnaire data	152 key stakeholders including community health workers, supervisors, nurses in charge, district health promoting officers completed questionnaires.	- Reach<br><br>- Effectiveness<br><br>- Adoption<br><br>- Implementation
Walker et al 2020 [76]	United Kingdom	HIC	ESCAPE-pain; an evidence-based intervention integrating education and exercise	Patients with knee / hip pain and osteoarthritis	R	P, I, C	RE-AIM	- Adoption of program by Academic Health	Routine data monitoring to measure outcomes for each of the	Outcome data from 3873 patients with osteoarthritis (hip or	- Reach<br><br>- Effectiveness

			for people with chronic knee and/or hip pain and osteoarthritis,<br><br>which promotes self-management to improve quality of life and function.					Science Networks<br><br>- Small team to support scale-up including project manager and administrative support<br><br>- National program manager and dedicated resource within each of 15 networks.	RE-AIM domains. Self-reported compliance with the ESCAPE-Pain core components	knee)	- Adoption<br><br>- Implementation<br><br>- Maintenance
Waqa et al 2013 [77]	Fiji	UM	A community-based intervention program: Healthy Youth Healthy Communities to increase the capacity of the<br><br>whole community, including churches, mosques, and temples, to promote healthy eating and regular physical activity, and to prevent unhealthy weight gain in adolescents aged 13–18 years	Adolescents	Prevention	P, I, C	n/a	- Capacity building<br><br>- Social marketing	Quantitative data collection using bespoke data collection form about intervention planning, delivery activities, process, dose, reach, people involved, frequency and associated resource use	Unclear	- Identification of successful objectives and success factors within broader action plan in relation to context
Zhou et al 2022 [78]	China	UM	Quality improvement program for acute coronary syndromes in hospitals	Patients with acute coronary syndrome	NCD	P, I, C	CFIR	- Implementation of an accreditation program<br><br>- Uniform guidance on pain centre organization and management<br><br>- Quality control system	Semi-structured interviews, partly individual partly focus-group discussions	Director and coordinators of multi-stage cluster sampled acute chest pain centres	- Barriers<br><br>- Facilitators<br><br>- Strategies to overcome barriers

* Navigation is an intervention modelled after existing evidence-informed interventions where client navigators provide individualized support for clients to access medical and social services, promote re-engagement in care, maintain medication adherence, and keep track of medical appointments. ** HIC, High-Income Country; LIC, Low-Income Country; LM, Low-Middle-Income Country; UM, Upper-Middle-Income Country; ^Scale-up dimensions: P, increasing population coverage; I, integrating services; and C, increasing comprehensiveness. Abbreviations: CFIR, Consolidated Framework for Implementation Research; RE-AIM, Reach, Effectiveness, Adoption, Implementation, and Maintenance; MRC, Medical Research Council; i-PARIHS, integrated-Promoting Action on Research Implementation in Health Services; NPT, Normalisation Process Theory; PRACTIS guide, PRACTical planning for Implementation and Scale-up;

CAS lens, complex adaptive system lens. [$]health outcome targeted by the CHI: NCD, noncommunicable diseases; CD, communicable diseases; MCH, maternal and child health; and R, rehabilitation.
