# Supplementary material for: Process Evaluations for the Scale-Up of Complex Interventions – a Scoping Review
Source: Int J Integr Care. 2024 Nov 8;24(4):6. doi: 10.5334/ijic.7600 (PMC11546072; doi:10.5334/ijic.7600)
Supplement: Supplementary Material 3. — Supplementary data – Table 1. [file ijic-24-4-7600-s3.pdf]

Supplementary data – Table 1

Studies (n=35)	Description of CHI	Country's income classification	Country	Scale-up or implementation framework used	Scale-up or implementation framework used	Study design	Study population
Ajayi et al 2008 (27)	Home management of malaria	LM	Nigeria (South-West)	Feasibility and community perception	feasibility and community perception	quasi-experimental study	40 communities at baseline
Arrossi et al 2017 (17)	HPV self-collection	H	Argentina (Jujuy Province)	RE-AIM (7) and adaptation of health system framework	RE-AIM and adaptation of health system framework	RCT	women beneficiaries
Bennett et al 2017 (43)	Community-based intervention	L, L, LM	Afghanistan, Uganda, Bangladesh	CFIR (44)	CFIR	n/a	3 implementation research studies
Bottorff et al 2021 (45)	Intervention targeting obesity in rth America	H	Canada	RE-AIM (7)	RE-AIM framework	n/a	health promotion healthy together
Brunton et al 2022 (46)	Scale-up of ABC care bundle for stroke	H	England (Greater Manchester)	i-PARIHS (47)	i-PARIHS	quality improvement project	scaled-up to two hyperacute stroke units
Cintyamena et al 2021 (48)	Scaling up polio eradication	LM to UM in the period (UM in 2019)	Indonesia	Barker, Reid and Schall (2016) four steps to scaling up (49)	Barker, Reid and Schall (2016) four steps to scaling up	n/a	polio vaccine recipients
El Joueidi et al 2021 (18)	Digital health tool: WelTel	L, H	East Africa, Canada	CFIR (44)	mCFIR	digital health tool after RCT	n/a
Fikree et al 2020 (50)	long-acting reversible contraceptive (LARC) uptake in YFS	L	Ethiopia	Their own one-stop-shop youth-friendly service (YFS) delivery model based on ExpandNet, Yamey, CAS lens, and Breastfeeding Gear model (51–54)	their own one-stop-shop YFS delivery model	n/a	182 YFS units in 4 regions
Fischer et al 2019 (32)	NurseConnect supporting nurses and midwives in maternal and child health	UM	South Africa	none	none	n/a	18 facilities randomly
Georgeu et al 2012 (19)	Streamlining Tasks and Roles to Expand Treatment and Care for HIV (STRETCH) Trial task-shifting for	UM	South Africa	Assessing fidelity of the implementation of the intervention under study, describing processes, relationships and	assessment of the fidelity of the implementation of the intervention under study	RCT	task-shifting for HIV/AIDS care from physicians to nurses tested at two kinds of antiretroviral therapy sites, one nurse-led

	HIV/AIDS care from physicians to nurses			contexts involved in CHI delivery, identifying barriers/facilitators with the aim to address black box problems (theory-based PE alongside an RCT of printed educational materials to improve referral and prescribing practices (55)			and one physician-led
Gong et al 2021 (25)	Primary-care-based integrated mobile health intervention (SINEMA)	UM	China (rural)	RE-AIM (7) framework and the MRC process evaluation framework	both the RE-AIM framework and the MRC process evaluation framework	1-year-cluster-RCT	25 rural villages, 637 patients
Hargreaves et al 2010 (26)	Intervention with Microfinance for AIDS and Gender Equity (IMAGE) combines microfinance	UM	South Africa	none	none	cluster-RCT	430 female clients
Holroyd-Leduc et al 2019 (34)	Mobilization of Vulnerable Elders (MOVE) intervention	H	Canada (Alberta province)	Knowledge to Action (KTA) cycle. (56)	Knowledge to Action (KTA) cycle. pragmatic, quasi-experimental un-blinded interrupted time series (ITS) design	n/a	four community hospitals
Hunt et al 2020 (24)	Football Fans in Training (FFIT) program	H	Started in the UK, scaled-out to Australia, Canada, New Zealand, England, other EU countries	PRACTIS guide (57)	PRACTIS guide	RCT	men aged 35–65 who are at increased risk of future ill-health because of their body size (BMI > 28 kg/m2)
Irungu et al 2021 (58)	PreP integration into routine clinical practice	LM	Kenya	Framework for Reporting Adaptations and Modifications-Enhanced (FRAME) (59)	Framework for Reporting Adaptations and Modifications-Enhanced (FRAME)	prospective implementation study	25 high volume public HIV care clinics
Kauchali et al 2022 (29)	Management of severe acute malnutrition (SAM)	UM, LM, L, LM	South Africa, Bolivia, Malawi, and Ghana	none	n/a	case studies	4 country studies of scaling up implementation of WHO guidelines for improving the inpatient management of SAM

Lafferty et al 2021 (20)	The Test Treat ANd GO (TTANGO) trial	H	Australia (remote Aboriginal and Torres Strait Islander communities)	Milat et al.'s (9) Increasing the scale of population health interventions: A Guide	Milat et al.'s [ref] Increasing the scale of population health interventions: A Guide	RCT	15 HCWs and 5 managers
Liu et al 2020 (60)	10-week Mind, Exercise, Nutrition … Do it! (MEND) British Columbia (B.C.) program	H	Canada	RE-AIM (7)	RE-AIM	prospective pragmatic implementation evaluation	27 sites
Low et al 2021 (38)	Enhanced Primary Health Care (EnPHC) Initiative	UM	Malaysia	Fidelity, feasibility, adaptation and perceived benefit (61,62)	fidelity, feasibility, adaptation and perceived benefit	complex intervention package	20 participating EnPHC clinics
MacGregor et al 2018 (63)	Adherence club antiretroviral therapy	UM	South Africa (Western Cape province)	CAS lens (53)	CAS lens	adherence club intervention	City of Cape Town health department
Mary et al 2019 (64)	Postpartum haemorrhage (PPH) interventions	L	Niger	none	none	national PPH intervention scaled up focusing on three clinical interventions	69 health facilities
Morgan et al 2022 (65)	Rural memory clinics	H	Canada	none	none	qualitative cross-sectional retrospective study	four interdisciplinary rural memory clinic teams
Mulat et al 2022 (36)	Community-based health insurance (CBHI)	L	Ethiopia	none	none	community-based health insurance	13 woredas (districts)
Nsangi et al 2019 (35)	Informed health choices (IHC) project	L	Uganda	none	none	informed health choices trial	60 schools
Pichon et al 2021 (66)	Project PrIDE	H	USA	none	none	pre-exposure prophylaxis (PrEP) navigation*	2018: nine PrEP navigators 2019: five of the original navigators
Puchalski Ritchie LM et al 2021 (67)	Training rounds to support patient tuberculosis (TB) treatment adherence	L	Malawi	RE-AIM (7)	RE-AIM	RCT	4 districts in S/E zone of Malawi
Rhodes et al 2020 (68)	Community-Based Participatory Research	H	USA	none	n/a	n/a	beneficiaries
Sako et al 2018 (42)	Nutrition education	L	Ethiopia	Yamey framework (52)	Yamey framework	grain bank	Eight study sites
Sivakumar et al 2022 (31)	Patient decision aid tool	H	Canada	NPT (69)	NPT	n/a	Ontario PHC system
Tonnon et al 2016 (22)	Lifestyle intervention Health Under Construction	H	The Netherlands	Steckler and Linnan framework (70) and additional components	Steckler and Linnan framework	RCT	Dutch construction workers
Walker et al 2020 (23)	ESCAPE-pain	H	UK	RE-AIM (7)	RE-AIM	complex evidence-based intervention	110 clinical and non-clinical sites reaching

						underpinned by an RCT and an economic evaluation	over 9000 people with osteoarthritis
Waqa et al 2013 (28)	Healthy Youth Healthy Communities	UM	Fiji	n/a	n/a	multi-faceted community-based interventions in a quasi-experimental design	adolescents aged 13–18 years
Zhou et al 2022 (71)	quality improvement program for acute coronary syndrome	UM	China	CFIR (44)	CFIR	n/a	patients with IHD - in National Chest Pain Centers Program (NCPCP)
Renju et al 2010 (41)	Model youth-friendly services for adolescent sexual and reproductive health intervention	L	Tanzania (rural)	none	n/a	multi-component adolescent sexual and reproductive health community-randomized trial	4 districts, there are 6 hospitals, 24 health centres and 154 dispensaries
Renju et al 2010 (39)		L	Tanzania (northern)	Framework for Reporting Adaptations and Modifications-Enhanced (FRAME) (59)	n/a		4 of Mwanza's 8 districts
Renju et al 2011 (40)		L	Tanzania (northern)		n/a		schools
McLaughlin 2021 (37)	Physical Activity 4 Everyone (PA4E1)	H	Australia and New Zealand		FRAME	type III hybrid implementation-effectiveness scale-up trial	low-socioeconomic secondary schools (n=49)

Legend: *Navigation is an intervention modelled after existing evidence-informed interventions where client navigators provide individualized support for clients to access medical and social services, promote re-engagement in care, maintain medication adherence, and keep track of medical appointments; CFIR, Consolidated Framework for Implementation Research; RE-AIM, Reach, Effectiveness, Adoption, Implementation, and Maintenance; MRC, Medical Research Council; i-PARIHS, integrated-Promoting Action on Research Implementation in Health Services; PRACTIS guide, PRACTical planning for Implementation and Scale-up; CAS lens, complex adaptive system lens
